# TAK1 and IKK2, novel mediators of SCF-induced signaling and potential targets for c-Kit-driven diseases

**DOI:** 10.18632/oncotarget.5008

**Published:** 2015-09-01

**Authors:** Sebastian Drube, Franziska Weber, Christiane Göpfert, Romy Loschinski, Mandy Rothe, Franziska Boelke, Michaela A. Diamanti, Tobias Löhn, Julia Ruth, Dagmar Schütz, Norman Häfner, Florian R. Greten, Ralf Stumm, Karin Hartmann, Oliver H. Krämer, Anne Dudeck, Thomas Kamradt

**Affiliations:** ^1^ Institut für Immunologie, Universitätsklinikum Jena, Jena, Germany; ^2^ Georg-Speyer-Haus, Institute for Tumorbiology and Experimental Therapy, Frankfurt, Germany; ^3^ Institut für Pharmakologie, Universitätsklinikum Jena, Jena, Germany; ^4^ Gynäkologische Molekularbiologie, Klinik für Frauenheilkunde und Geburtshilfe, Jena, Germany; ^5^ Klinik und Poliklinik für Dermatologie und Venerologie, Universität zu Köln, Köln, Germany; ^6^ Institut für Toxikologie, Universitätsmedizin Mainz, Mainz, Germany; ^7^ Institute for Immunology, Technische Universität Dresden, Medical Faculty Carl Gustav Carus, Dresden, Germany

**Keywords:** mast cells, TAK1-IKK2 activation, c-Kit-Lyn-TAK1-IKK2 complex, mitogenic signaling, NF-κB-activation

## Abstract

NF-κB activation depends on the IKK complex consisting of the catalytically active IKK1 and 2 subunits and the scaffold protein NEMO. Hitherto, IKK2 activation has always been associated with IκBα degradation, NF-κB activation, and cytokine production. In contrast, we found that in SCF-stimulated primary bone marrow-derived mast cells (BMMCs), IKK2 is alternatively activated. Mechanistically, activated TAK1 mediates the association between c-Kit and IKK2 and therefore facilitates the Lyn-dependent IKK2 activation which suffices to mediate mitogenic signaling but, surprisingly, does not result in NF-κB activation. Moreover, the c-Kit-mediated and Lyn-dependent IKK2 activation is targeted by MyD88-dependent pathways leading to enhanced IKK2 activation and therefore to potentiated effector functions. In neoplastic cells, expressing constitutively active c-Kit mutants, activated TAK1 and IKKs do also not induce NF-κB activation but mediate uncontrolled proliferation, resistance to apoptosis and enables IL-33 to mediate c-Kit-dependent signaling. Together, we identified the formation of the c-Kit-Lyn-TAK1 signalosome which mediates IKK2 activation. Unexpectedly, this IKK activation is uncoupled from the NF-κB-machinery but is critical to modulate functional cell responses in primary-, and mediates uncontrolled proliferation and survival of tumor-mast cells. Therefore, targeting TAK1 and IKKs might be a novel approach to treat c-Kit-driven diseases.

## INTRODUCTION

The IKK complex consists of IKK1/2 and NEMO. IKK1 and 2 are the catalytically active components whereas NEMO is the regulatory scaffold protein of the IKK complex [1–3]. Stimulation of receptors of the TLR/IL-1 (TIR) - or TNFR superfamily results in a poly-ubiquitinylation-dependent phosphorylation of the serine (S) residue motive, S177/S181 of IKK2. This motive is located in the activation loop of IKK2 [4, 5] and is pivotal for IKK2 activation [5]. TAK1 is proposed as the upstream IKK2 kinase which mediates S177/S181 phosphorylations and therefore activation of IKK2 [6]. The consequence of IKK2 activation is the phosphorylation of the NF-κB inhibitor IκBα which is ubiqiutinylated and proteasomal degraded. Therefore, NF-κB is released, translocates into the nucleus and induces a cytokine production [7]. Recent publications suggests that receptor tyrosine kinases (RTKs) also activate NF-κB via the IKK complex [8, 9].

The RTK, c-Kit is critical for differentiation of cells of the haematopoietic system [10–12]. Mast cell differentiation, survival and proliferation also depend on c-Kit and on the resulting STAT3/5, p38, and Phosphoinositide-3-kinase (PI3K) activation [13–18]. However, expression of constitutively active c-Kit mutants leads to persistently activated STAT3/5, MAP-kinases, and PI3Ks and results in the development of mastocytosis or mast cell tumors [19–23].

The IKK complex and NF-κB are important to mediate the effector functions of mature mast cells stimulated with ligands of the TIR superfamily (e.g. IL-33R; [24]) or by crosslinking of the Fcε receptor I [FcεRI; [25]). Recent data suggest that IKKs are also crucial to induce NF-κB-dependent tumorigenesis [26].

We show that stimulation of BMMCs with SCF, the overexpression of wt c-Kit or the expression of constitutively active c-Kit mutants mediates activation of IKK2. Surprisingly, the c-Kit-mediated IKK2 activation does not lead to NF-κB activation but is critical for differentiation, proliferation and survival of primary- and tumor-mast cells. Therefore, the c-Kit-mediated IKK2 activation regulates mast cell effector functions and might be a novel approach to treat c-Kit-driven diseases.

## RESULTS

### SCF induces IKK2 activation

**Figure 1:**
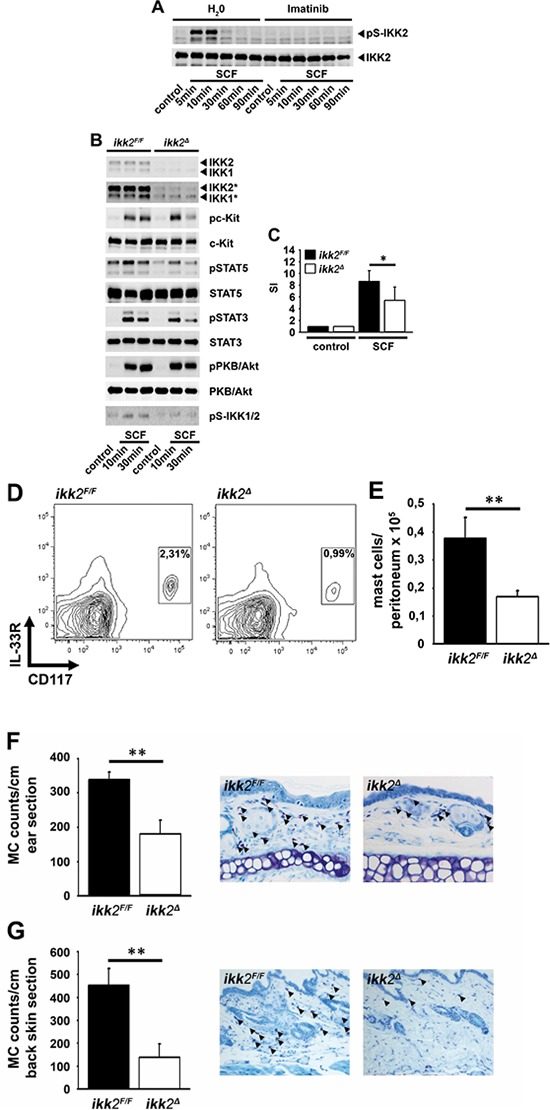
SCF induces the activation of IKK2 **A.** BMMCs were pre-treated with H_2_O (vehicle) or imatinib (5 μM) and stimulated with SCF (50 ng/ml). Lysates were analyzed by western blotting. **B, C.** BMMCs from *ikk2^F/F^* or *ikk2^Δ^* mice were stimulated with SCF (50 ng/ml). Lysates were analyzed by western blotting (B; * longer exposure) or cells were probed with [H^3^]-thymidine and analyzed by *β*-counting (C). **D, E.** Peritoneal fluid from *ikk2^F/F^* or *ikk2^Δ^* mice was collected and analyzed for the presence of mast cells by flow cytometry. Contour graphs are shown, characterizing mast cells as CD117^+^/IL-33R^+^ cells (D). Total numbers of CD117^+^/IL-33R^+^ mast cells per peritoneum were calculated (this experiment was performed two times; *ikk2^F/F^* mice, *n* = 3; *ikk2^Δ^* mice, and *n* = 3) (E). **F, G.** Mast cell numbers were counted per ear (F) or back skin section (G) over a length of 1 cm. Mean of 3 sections per mouse (*ikk2^Δ^* mice, *n* = 4; *ikk2^Δ^* mice, *n* = 6) was calculated ± SD.

Aiming to investigate the SCF-induced signaling in BMMCs we unexpectedly found that SCF induces IKK2 activation which was blocked by the c-Kit inhibitor imatinib (Figure 1A). To delineate the role of IKK2 in the SCF-induced signaling and effector functions, we used IKK2 deficient BMMCs from inducible *ikk2^Δ^* mice [27] and from cre^−^, *Ikk2^F/F^* control mice, as well as the IKK-inhibitor VII [28, 29]. We focused on the SCF-induced STAT and PI3K activation, since inactivation of these signaling cascades, but not MAPKs (Supplementary Figures S1A–S1C) compromises mitogenic mast cell responses [17, 18, 30, 31].

The reduced expression of IKK2 in *ikk2^Δ^* BMMCs (Figure 1B) does not affect surface expression of c-Kit compared to control *ikk2^F/F^* BMMCs (Supplementary Figure S1D). The SCF-induced PKB/Akt activation was unperturbed, whereas the activation of STAT3/5 was reduced in *ikk2^Δ^* compared to *ikk2^F/F^* BMMCs (Figure 1B). To confirm these results we used the IKK-inhibitor VII [32]. We pre-incubated BMMCs with different IKK-inhibitor VII concentrations and stimulated with SCF. We found that the IKK-inhibitor VII does not affect the SCF-induced activation of PKB/Akt but strongly reduced the activation of STAT3/5 and proliferation even at very low concentrations (0,5–0,1 μM) (Supplementary Figures S1E and S1F). Compared to the results obtained with *ikk2^Δ^* BMMCs, these data show that IKK-inhibitor VII treatment or the reduced IKK2 expression affected the same SCF-induced signaling pathways. Therefore, it can be assumed that the IKK-inhibitor VII preferentially blocks IKK activation in mast cells. Given that STATs are critical for mast cell differentiation, we examined whether IKK2 contributes to proliferation and differentiation. As shown in Figures 1C–1G, we found a slightly reduced proliferation (Figure 1C) and a profound reduction of mast cell numbers in the peritoneal fluid (Figures 1D and 1E), the ear- (Figure 1F) and back-skin of *ikk2^Δ^* mice (Figure 1G) compared to control *ikk2^F/F^* mice. These data show that the SCF-induced IKK2 activation mediates differentiation via STAT3/5.

### IKKs mediate proliferation and cell survival of tumor mast cells

**Figure 2:**
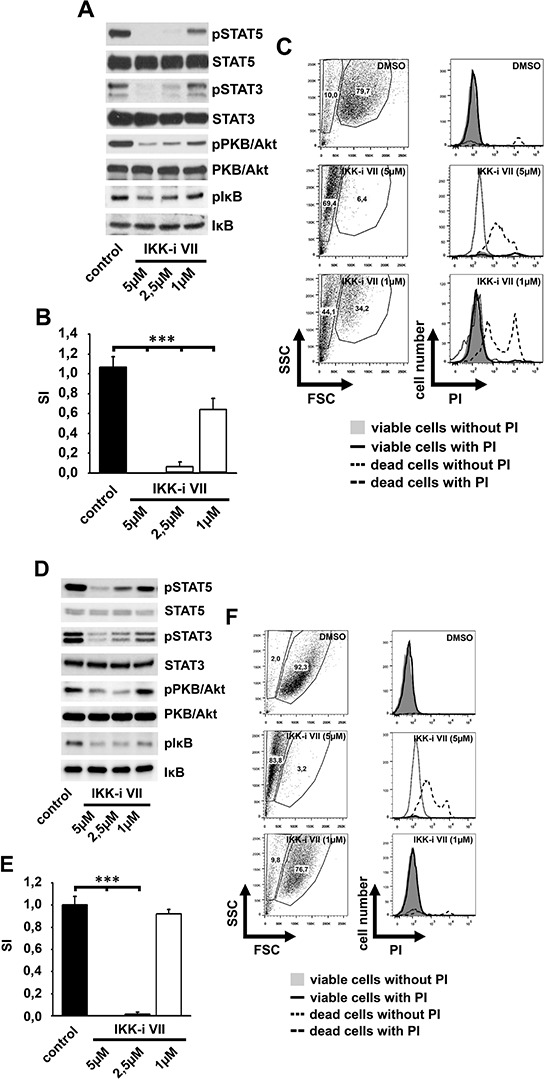
IKK inhibition induces cell death in HMC-1.1 and HMC-1.2 cells HMC-1.1 cells **A–C. D–F.** were treated with different concentrations of the IKK-inhibitor VII. Cells were lysed and analyzed by western blotting (A, D), were probed with [H^3^]-thymidine and analyzed by *β*-counting (B, E) or were treated with PI after 48 h and analyzed by flow cytometry (C, F).

We next investigated whether IKKs also contribute to the signaling induced by constitutively active c-Kit mutants. Therefore, we used HMC-1.1 (expressing the imatinib-sensitive V560G c-Kit mutant) and HMC-1.2 cells (expressing the imatinib-insensitive D816V/V560G c-Kit mutant) [33]. Indeed, incubation of HMC-1.1 cells (Figures 2A–2C) or HMC-1.2 cells (Figures 2D–2F) with the IKK-inhibitor VII blocked the constitutive phosphorylation of STAT3/5, IκBα and also of PKB/Akt (Figures 2A, 2D). Consequently, the IKK-inhibitor VII blocked the proliferation (Figures 2B, 2E) by inducing cell death (Figures 2C, 2F) in both, HMC-1.1 and HMC-1.2 cells and therefore overcomes imatinib resistance.

**Figure 3:**
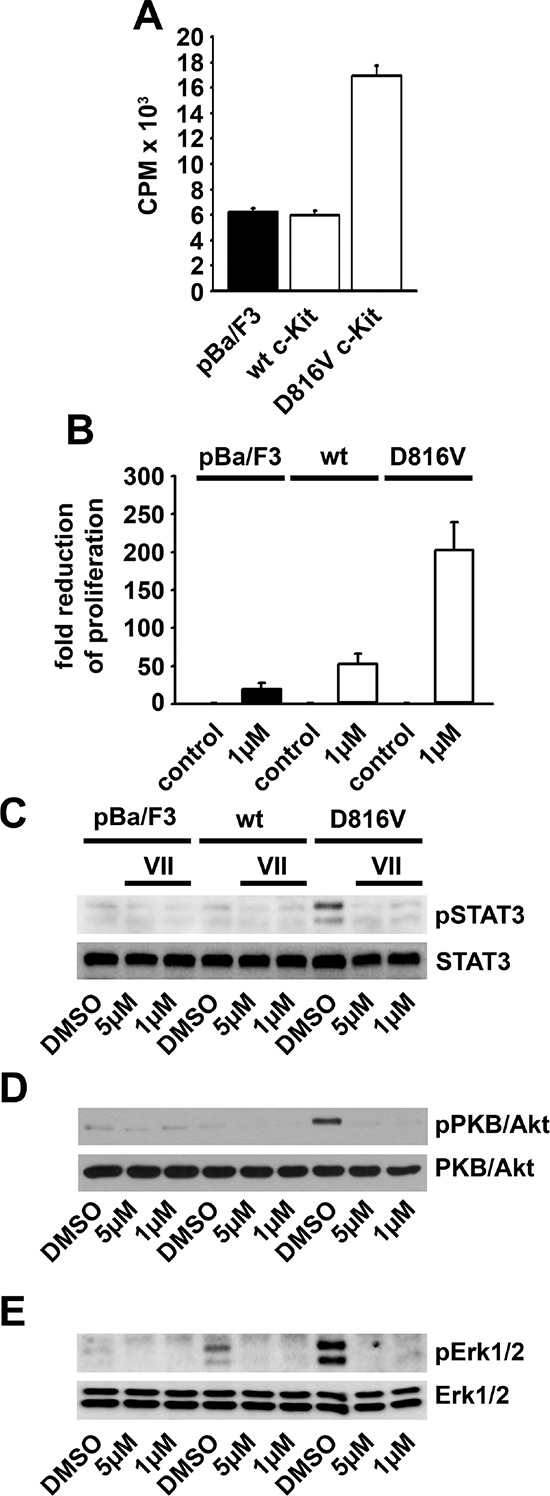
Expression of the D816V c-Kit mutant mediates IKK-dependent transformation **A.** Parental Ba/F3 cells (pBa/F3), wt c-Kit-expressing Ba/F3 cells (wt) or D816V c-Kit-expressing Ba/F3 cells (D816V) were probed with [H^3^]-thymidine and analyzed by *β*-counting. **B.** PBa/F3, wt or D816V cells were treated with the IKK-inhibitor VII. Cells were probed with [H^3^]-thymidine and analyzed by *β*-counting. **C–E.** PBa/F3, wt or D816V cells were treated with the IKK-inhibitor VII, lyzed and analyzed by western blotting.

Next, we investigated the transforming potential of the D816V c-Kit mutant and its dependency on IKKs. Confirming others [34], expression of the D816V Kit mutant, but not of wt c-Kit potentiated the basal proliferation of Ba/F3 cells (Figure 3A). Pre-treatment with the IKK-inhibitor VII reduced the basal proliferation of D816V c-Kit-expressing Ba/F3 cells for about 200 fold whereas the proliferation of parental Ba/F3 or wt c-Kit-expressing Ba/F3 cells was reduced up to 50 fold (Figure 3B). This shows that D816V c-Kit-expressing Ba/F3 cells are up to four times more sensitive to the IKK-inhibitor VII than parental Ba/F3 or wt c-Kit-expressing Ba/F3 cells.

We further examined the induced signaling pathways in these Ba/F3 cell clones. In parental Ba/F3 we could not detect the activation of STATs, PKB/Akt and MAP-kinases (Figures 3C–3E). In wt c-Kit-expressing Ba/F3 cells, the activation of Erk1/2 but not of PKB/Akt and STATs was slightly increased (Figures 3C–3E). In contrast, in D816V c-Kit-expressing Ba/F3 cells we found a strong activation of Erk1/2, PKB/Akt and STAT3 which were blocked by the IKK-inhibitor VII (Figures 3C–3E). These data indicate that constitutively active c-Kit mutants mediate IKK-dependent mitogenic signaling.

### Src-family kinases (SFKs) mediate IKK2 and STAT3/5 activation

**Figure 4:**
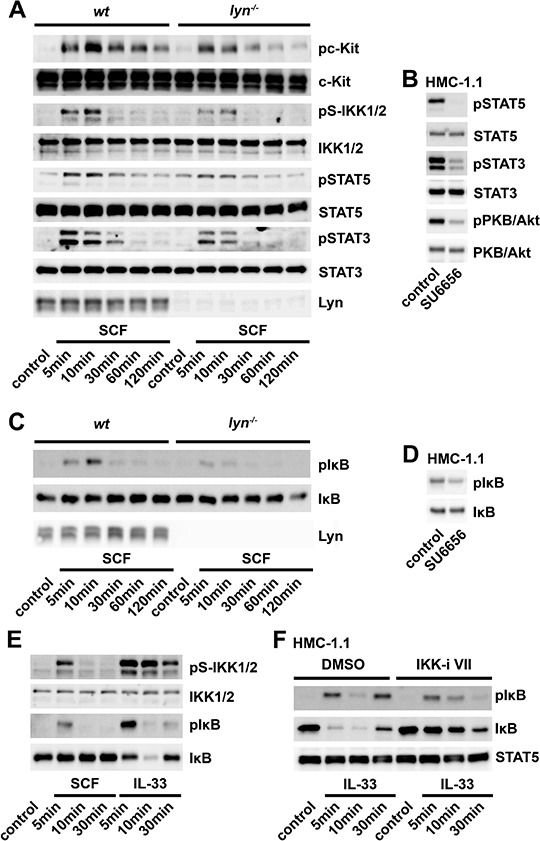

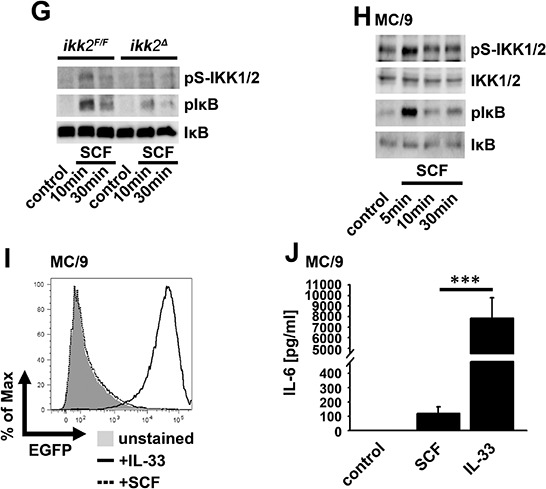
The SCF-induced IKK2 activation depends on SFKs but does not induce NF-κB activation **A.** Wt- or *lyn^−/−^* BMMCs were stimulated with SCF (50 ng/ml). Lysates were analyzed by western blotting. **B.** HMC-1.1 cells were treated with SU6656, lyzed and analyzed by western blotting. **C.** Wt- or *lyn^−/−^* BMMCs were stimulated with SCF (50 ng/ml). Lysates were analyzed by western blotting. **D.** HMC-1.1 cells were treated with SU6656, lyzed and analyzed by western blotting. E. BMMCs were stimulated with SCF or IL-33 (both 50 ng/ml) and lysates were analyzed by western blotting. F. HMC-1.1 cells were treated with the IKK-inhibitor VII and stimulated with IL-33. Lysates were analyzed by western blotting. **G.** *Ikk2^F/F^* or *ikk2^Δ^* BMMCs were stimulated with SCF (50 ng/ml). Lysates were analyzed by western blotting. **H.** NF-κB-EGFP-MC/9 cells were stimulated with SCF (50 ng/ml). Lysates were analyzed by western blotting. **I, J.** NF-κB-EGFP-MC/9 cells were stimulated with SCF or IL-33 (both 50 ng/ml). Cells were analyzed for EGFP production by flow cytometry (I) or supernatants were analyzed by ELISA for IL-6 production (J).

Now, we focused on the mechanism leading to IKK2 activation in BMMCs. SFKs regulate most of the SCF-induced signaling pathways [35]. Pre-incubation of the SFK inhibitor SU6656 impaired the activation of c-Kit, IKK2 and STATs but enhanced the activation of PKB/Akt and did not affect the proliferation (Supplementary Figures S2A and S2B). Next, we tested the role Jak2 known as an upstream kinase of STATs [36]. Shivakrupa *et al*., showed that Lyn is essential for the SCF-induced STAT activation in BMMCs [37]. Additionally we found that the Jak2 inhibitor AG490 did not influence the SCF-induced proliferation (Supplementary Figure S2C). This confirms that SFKs but not Jak2 are required for the SCF-induced STAT activation and the resulting proliferation in BMMCs. Consequently, we suggest that SFKs positively regulate the IKK2-STAT pathway and negatively regulate the PI3K-PKB/Akt pathway. Lyn negatively regulates the PI3K-PKB/Akt pathway and the resulting proliferation in mast cells [13, 31]. Thus, we speculated that Lyn also mediates activation of the IKK2-STAT pathway. To test this hypothesis we used *lyn^−/−^* BMMCs. Indeed, activation of c-Kit, IKK2 and STATs were reduced (Figure 4A), whereas the activation of PKB/Akt and the resulting proliferation were enhanced (Supplementary Figures S2D and S2E) in *lyn^−/−^*, compared to wt BMMCs. Therefore, inactivation of the mitogenic IKK2-STAT signaling is compensated by upregulation of the PKB/Akt signaling in *lyn^−/−^* BMMCs. This shows that Lyn positively regulates the c-Kit-IKK2-STAT pathway and negatively regulates the PI3K-PKB/Akt pathway.

Given that Ca^2+^ is required for IKK2 activation [38], we tested the role of the PLCγ-mediated Ca^2+^ mobilization (Supplementary Figure S3A) in the SCF-induced IKK2-STAT signaling. Pre-treatment with the PLC inhibitor U-73122 or the Ca^2+^ chelator BAPTA-AM reduced the SCF-induced activation of the c-Kit-IKK2-STAT pathway whereas the proliferation was not affected (Supplementary Figures S3B–S3E).

Next, we examined the role of SFKs in the IKK-dependent activation of STATs in HMC cells. Confirming and extending others [39] pre-treatment of HMC-1.1 cells with SU6656 blocked the phosphorylation of STAT5/3 and PKB/Akt (Figure 4B). These data show that SFKs, as Lyn contribute to activation of the c-Kit-IKK2-STAT pathway in mast cells.

### The c-Kit-mediated IKK2 activation does not result in NF-κB activation

IKK2 typically mediates NF-κB activation. Thus, we determined whether SCF or constitutively active c-Kit mutants induce IκBα phosphorylation, degradation and NF-κB activation. Confirming that IKK2 activation depends on Lyn-, PLCγ- and Ca^2+^, the SCF-induced IκBα phosphorylation was blocked in *lyn^−/−^* BMMCs (Figure 4C) or BMMCs treated with U-73122 or BAPTA-AM (Supplementary Figures S3F and S3G). Complementing these findings, treatment of HMC-1.1 cells with the SFK inhibitor SU6656 reduced the phosphorylation of IκBα (Figure 4D). However, neither SCF nor the expression of constitutively active c-Kit mutants influenced the stability of IκBα (Figures 4C and 4D, Supplementary Figures S3F and S3G) suggesting defective IKK2-IκBα signaling in our BMMCs or HMC-1.1 cells.

To test the IKK2-IκBα signaling we stimulated with IL-33 a typical inducer of the MyD88- and IKK2-dependent canonical NF-κB signaling [40]. In contrast to SCF, IL-33 induces a strong activation of IKK2, which results in phosphorylation and degradation of IκBα (Figure 4E) in BMMCs. Furthermore, the IL-33- but not the SCF-induced effector functions were blocked in *myd88^−/−^* BMMCs (Supplementary Figures S4A and S4B). Showing the intact IKK2-IκBα signaling in HMC-1.1 cells, stimulation with IL-33 induced an IKK-inhibitor VII-sensitive IκBα phosphorylation and degradation (Figure 4F). We next determined whether alternative pathways, independently of IKK2, mediate the SCF-induced IκBα phosphorylation in BMMCs. Therefore, we used *ikk2^Δ^* BMMCs or the IKK-inhibitor VII. In both cases the SCF-induced IκBα phosphorylation was blocked (Figure 4G, Supplementary Figure S4C). Together these results show that the IKK2-IκBα signaling is intact, and is activated by SCF and IL-33 whereas only IL-33 stimulation induced an IKK activation which is strong enough to induce IκBα degradation.

Finally, we determined the capacity of SCF to induce NF-κB activation. Therefore, we used NF-κB-EGFP-MC/9 cells [41]. In these cells we also found the SCF-induced IKK2 activation but no IκBα degradation (Figure 4H). Compared to IL-33, SCF did not induce EGFP expression or a strong cytokine production (Figures 4I and 4J) indicating that SCF either induces a very weak or no NF-κB activation.

Why does SCF not induce IκBα degradation and NF-κB activation? We found that SCF in presence of cycloheximide, a protein-biosynthesis inhibitor, induces IκBα degradation **(data not shown)**. Therefore, in presence of ongoing IκBα re-synthesis, the SCF-induced IκBα degradation is quantitatively not sufficient to induce a net loss of IκBα.

Together, we found that neither stimulation with SCF nor the expression of constitutively active c-Kit mediates IκBα degradation. Consequently, we assume that compared to IL-33, activated c-Kit mediates an alternative mechanism leading to a weak “subthreshold IKK2 activation” [28] which is insufficient to mediate complete NF-κB activation.

### SCF induces tyrosine phosphorylation of IKK2 in BMMCs

**Figure 5:**
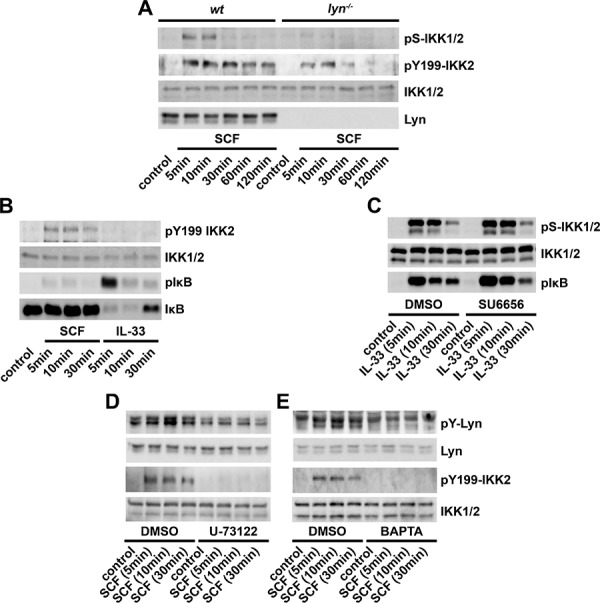
SCF induces phosphorylation of Y199-IKK2 in BMMCs **A.** Wt or *lyn^−/−^* BMMCs were stimulated with SCF (50 ng/ml). Lysates were analyzed by western blotting. **B.** BMMCs were stimulated with SCF or IL-33 (both 50 ng/ml). Lysates were analyzed by western blotting. **C.** BMMCs were pre-incubated with DMSO (vehicle) or SU6656 (5 μM) and stimulated with IL-33 (50 ng/ml). Lysates were analyzed by western blotting. **D, E.** BMMCs were pre-treated with DMSO (vehicle) or U-73122 (5 μM) (D) or BAPTA-AM (5 μM) (E) and stimulated with SCF (50 ng/ml). Lysates were analyzed by western blotting.

Huang *et al*. showed that in addition to phosphorylation of the S177/S181-IKK2 motive, also the SFK-mediated phosphorylation of the Y188/Y199-IKK2 motive is critical for IKK2 activation [42–44]. We found that the SCF-induced IKK2 activation is Lyn-dependent. Therefore, we speculated that SCF induces IKK2 activation via a Lyn-dependent phosphorylation of Y188/Y199-IKK2. Indeed, SCF induced the phosphorylation of Y199 which was reduced in *lyn^−/−^* BMMCs (Figure 5A). In contrast, IL-33 did neither induce the phosphorylation of Y199-IKK2 nor a SFK-dependent IKK2 activation (Figures 5B and 5C).

We identified the PLCγ-Ca^2+^ signaling as critical for the SCF-induced IKK2 activation. This indicated that the PLCγ-Ca^2+^ signaling activates Lyn and therefore mediates phosphorylation of Y199-IKK2. As shown in Figures 5D and 5E, treatment with U-73122 or BAPTA-AM blocked the activation of Lyn and the resulting phosphorylation of Y199-IKK2. Taken together, these data indicate that the SCF-induced IKK2 activation in BMMCs depends on the PLCγ-Ca^2+^-Lyn pathway leading to Y199-IKK2 phosphorylation and therefore IKK2 activation.

### Formation of the novel c-Kit-Lyn-TAK1 complex

**Figure 6:**
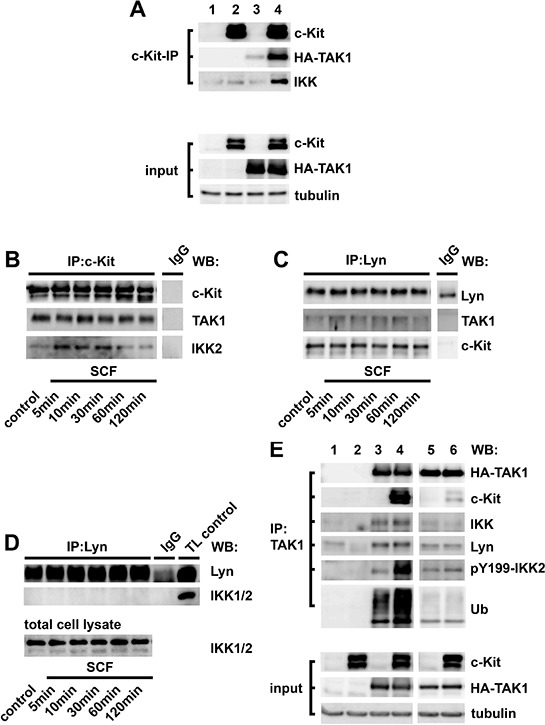
Formation of the c-Kit-Lyn-TAK1 complex **A.** HEK293T cells were transfected with pcDNA3.1 (empty vector, lane 1), pcDNA3.1-c-Kit (lane 2), pCMV-HA-wt-TAK1 (lane 3) or, pcDNA-c-Kit and pCMV-HA-wt-TAK1 together (lane 4). Lysates were subjected to a c-Kit-specific immunoprecipitation and the precipitates were analyzed by western blotting for c-Kit, HA-TAK1 and IKK2. As a control for equal expression we analyzed total lysates (input) for c-Kit, HA-TAK1 and tubulin. **B–D.** Lysates from SCF-stimulated (50 ng/ml) BMMCs were subjected to c-Kit- (B) or Lyn- (C, D) specific immunoprecipitations. Precipitates were analyzed by western blotting. Furthermore, we checked the expression of IKK2 in the total cell lysates by western blotting (D). **E.** HEK293T cells were transfected with pcDNA3.1 (empty vector) (lane 1), pcDNA3.1-c-Kit (lane 2), pCMV-HA-wt-TAK1 (lane 3) or pcDNA3.1-c-Kit and pCMV-HA-wt-TAK1 together (lane 4). Furthermore, we transfected pCMV-K63W-TAK1 alone (lane 5) or in combination with pcDNA3.1-c-Kit (lane 6). Lysates were subjected to a TAK1-specific immunoprecipitation and the precipitates were analyzed by western blotting. Furthermore, we also checked the equal expression of transfected proteins in the total cell lysates (input) by western blotting.

Now, we examined the molecular mechanism leading to IKK2 activation. First, we expressed c-Kit in HEK293T cells to test whether endogenously expressed IKK2 associates with c-Kit. Immunoprecipitation of overexpressed c-Kit did not result in co-precipitation of IKK2 (Figure 6A, lane 2) excluding association between c-Kit and IKK2. TAK1 is the upstream kinase of IKK2 [6]. Therefore, we speculated that additional TAK1 overexpression leads to co-precipitation of endogenous IKK2. Thus, we co-transfected HEK293T cells with TAK1 and c-Kit, performed a c-Kit-specific immunoprecipitation, and indeed detected IKK2 (Figure 6A, lane 4) showing that TAK1 mediates the interaction between c-Kit and IKK2. Confirming these results, we also found an association between c-Kit, TAK1 and IKK2 in c-Kit-specific immunoprecipitations from BMMC lysates (Figure 6B).

Given that Lyn is critical for IKK2 activation, we assumed that Lyn also joins the c-Kit-TAK1-IKK2 complex. In Lyn precipitates we detected c-Kit and TAK1 (Figure 6C) but not IKK2 (Figure 6D) indicating that Lyn interacts with c-Kit and TAK1 but not directly with IKK2.

Since, TAK1 was detectable in c-Kit and Lyn precipitates we assumed that TAK1 is the critical determinant for complex formation between c-Kit, IKK2 and Lyn. To determine the role of TAK1 we transfected HEK293T cells with c-Kit or TAK1 alone or both together. TAK1-specific immunoprecipitations confirmed the association between c-Kit and TAK1 (Figure 6E, compare lane 3 and 4). Furthermore, endogenous IKK2 and Lyn equally associates with TAK1 in cells either overexpressing TAK1 alone or TAK1 in combination with c-Kit (Figure 6E, compare lane 3 and 4) indicating that the Lyn-TAK1-IKK2 complex associates with c-Kit. However, only in cells co-overexpressing TAK1 and c-Kit, the phosphorylation of Y199-IKK2 and the TAK1 ubiquitinylation were potentiated (Figure 6E, compare lane 3 and 4). Given that Y199-IKK2 is a Lyn target, we tested whether Lyn activation is also increased in HEK293T cells co-overexpressing TAK1 and c-Kit. Indeed, in cells co-overexpressing both molecules, highly activated Lyn, co-precipitates with TAK1 and c-Kit (Supplementary Figure S5A, compare lane 3 and 4). Therefore, the preformed Lyn-TAK1-IKK2 complex is highly activated in presence of c-Kit.

Next, we investigated the role of the TAK1-activity in this complex formation. The TAK1 activity and its association with IKK2 strongly depend on Lysine 63 (K63) ubiquitinylation [45]. Overexpression of the inactive K63W-TAK1 mutant [45] abrogated the formation of the c-Kit-Lyn-TAK1-IKK2 complex and therefore blocked the phosphorylation of Y199-IKK2 (Figure 6E, compare lane 5 and 6). These data indicate that TAK1 ubiquitinylation mediates complex formation between c-Kit, IKK2 and Lyn. This expression model prompted us to investigate the role of TAK1 in BMMCs and HMC cells. Therefore, we used the TAK1 inhibitor 5Z-7-oxozeanol [46]. Pre-incubation of BMMCs with 5Z-7-oxozeanol reduced the activation of IKK2, STAT3 and STAT5 but not of PKB/Akt (Supplementary Figure S5B). In HMC cells 5Z-7-oxozeanol very potently blocked the activation of IKKs, STAT3/5 and also of PKB/Akt (Supplementary Figure S5C).

Here, we provide a mechanistical insight of the c-Kit-mediated IKK2 activation. Thereby, overexpression of c-Kit nearly mimics the effects occurring in SCF-stimulated cells or in cells expressing constitutively active c-Kit. From these experiments, we conclude that c-Kit forms a complex with TAK, SFKs and IKK2. The complex formation is thereby stabilized by TAK1 and results in a SFK-dependent IKK2 activation. Consequently, TAK1 is pivotal to induce the c-Kit-mediated activation of IKK2, STAT3/5 in primary- and tumor-mast cells.

### The conjoined activation of IKK2 by c-Kit- and MyD88-dependent pathways

**Figure 7:**
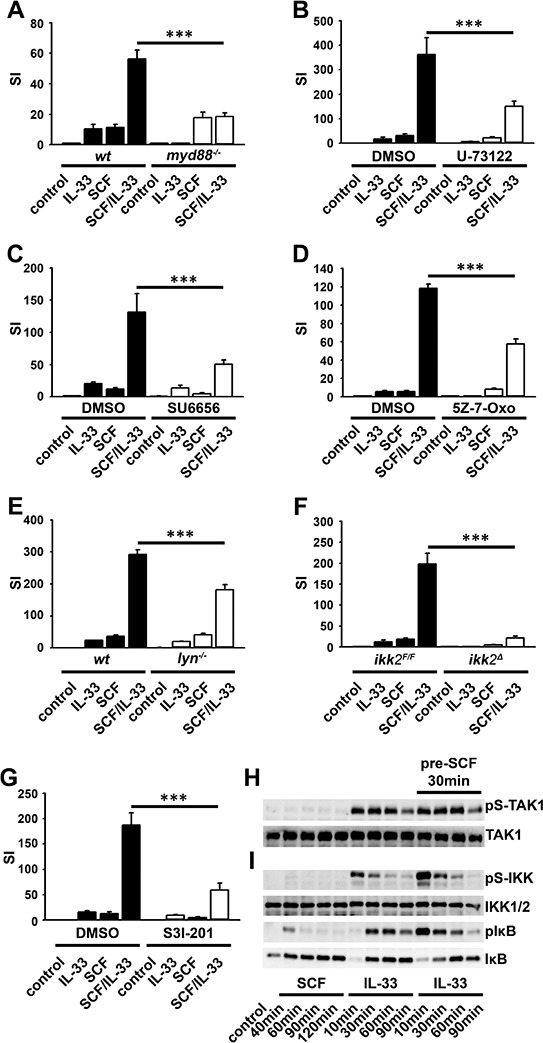

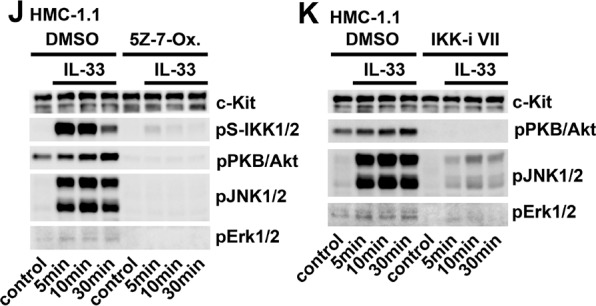
Activated c-Kit influences IL-33-induced signaling and effector functions in BMMCs and HMC cells Wt **A–G.**, *myd88^−/−^* (A), *lyn^−/−^* (E), *ikk2^F/F^*, *ikk2^Δ^* (F), or DMSO (vehicle) or inhibitor treated [5 μM U-73122 (B), 5 μM SU6656 (C), 0,5 μM 5Z-7-Oxozeanol (5Z-7-Oxo) (D), 50 μM S3I-201 (G)] BMMCs were stimulated with SCF (50 ng/ml) alone or in combination with IL-33 (50 ng/ml). Cells were probed with [H^3^]-thymidine and analyzed by *β*-counting (A–G). **H, I.** BMMCs were stimulated with SCF or IL-33 (both 50 ng/ml) or co-stimulated with both. Co-stimulation was performed by pre-incubation of the cells with SCF for 30min (pre-SCF 30min) followed by stimulation with IL-33 (for 10, 30, 60, 90 min). Lysates were analyzed by western blotting. **J, K.** HMC-1.1 cells were treated with the TAK1 inhibitor 5Z-7-oxozeanol (5Z-7-Ox.) (J) or the IKK-inhibitor VII (IKK-I VII) (K), and were stimulated with IL-33. Lysates were analyzed by western blotting.

Co-stimulation with SCF and IL-33 induces a potentiated IL-6 production [47] and proliferation in BMMCs. Both the potentiated proliferation (Figure 7A), and cytokine production (Supplementary Figure S6A) depends on MyD88.

Next, we investigated the role of the novel PLCγ-Ca^2+^-signaling which activates Lyn and therefore IKK2 in BMMCs. Pharmacological inhibition of PLCγ, SFKs, IKKs and TAK1 as well as Lyn and IKK2 deficiencies reduced the potentiated proliferation (Figures 7B–7F) and the IL-6 production (Supplementary Figures S6B–S6F). In contrast, inhibition of MAPKs did neither affect the potentiated proliferation (Supplementary Figures S6G and S6H) nor the potentiated IL-6 production in a adequate, biological relevant magnitude. (Supplementary Figures S6I and S6J).

IKK2 together with Lyn activate STAT3. Therefore, we examined the role of STAT3 in BMMCs co-stimulated with SCF and IL-33. Inhibition of STAT3 with S3I-201 [48] eradicated the synergistic effect of co-stimulation on proliferation (Figure 7G) but did not affect the potentiated IL-6 production (Supplementary Figure S6K).

Finally, we determined the effect of co-stimulation (SCF and IL-33) on the activation of TAK1 and IKK2. Single-stimulation with SCF or IL-33 induced the activation of TAK1 (Figure 7H) and IKK2 (Figure 7I). Co-stimulation did not increase the activation of TAK1 (Figure 7H) but enhanced the activation of IKK2 and delayed the IκBα re-synthesis (Figure 7I). These data shows that the SCF-induced and Lyn-dependent IKK2 is targeted by MyD88-dependent pathways. This leads to enhanced IKK2 activity which results in potentiated, NF-κB-dependent cytokine production, and in STAT-dependent potentiated proliferation.

### c-Kit supports IL-33-induced and TAK1- and IKK2-dependent signaling in HMC cells

In contrast to BMMCs, IL-33 induced a c-Kit-dependent activation of Erk1/2, JNK1/2 and PKB/Akt in HMC cells and in BMMCs generated from *kit^D814V^* tg mice [19, 47]. Here, we investigated whether TAK1 and IKK2 contribute to IL-33-induced and c-Kit-dependent signaling in HMC-1.1 cells. As shown in Figures 7J and 7K pre-incubation with the TAK1 inhibitor 5Z-7-oxozeanol or with the IKK-inhibitor VII blocked the IL-33-induced activation of PKB/Akt, JNK1/2 and Erk1/2. This shows that in contrast to BMMCs, constitutively active c-Kit together with TAK1 and IKK2 mediate the IL-33-induced activation of PKB/Akt, JNK1/2 and Erk1/2. Therefore, constitutively active c-Kit controls IL-33-induced and TAK1- and IKK-dependent signaling and effector functions.

## DISCUSSION

Here, we show that SCF and constitutively active c-Kit mutants activate TAK1 and IKK2. In contrast to TAK1 and IKK2 activation induced by antigen receptors, TIR- and TNFR superfamily family members [49–51], the c-Kit-mediated TAK1 and IKK2 activation is weak and results in IκBα phosphorylation but not in IκBα degradation and NF-κB activation. Instead, the c-Kit-mediated TAK1 and IKK2 activation induces STAT-dependent differentiation, proliferation and survival of primary- and tumor-mast cells.

Our findings differ from an earlier publication by Wu *et al*., who reported that SCF induces IκBα phosphorylation and degradation in BMMCs. In a later paper, the same group reported that SCF induces IκBα phosphorylation but not degradation in BMMCs which is in accordance with our data. The reason for these different findings remains unknown [52, 53]. We consistently found IκBα phosphorylation without degradation upon stimulation with SCF. Therefore, the relevant questions are: how does c-Kit mediate TAK1 and IKK2 activation and what is the difference of the IKK2 activation induced by TIR family members such as the IL-33R?

Both, c-Kit and IL-33R induce the activation of TAK1 and IKK2. Given that c-Kit does not induce IκBα degradation and NF-κB activation, this indicates that critical components for IκBα degradation (e.g. activation of MyD88-IRAK4-Traf6 signaling module) are not activated. Compared to IL-33R, c-Kit does not mediate the MyD88- and IRAK4-dependent **(data not shown)** pathways excluding the canonical pathway for IKK2 activation. Thus, we assumed an alternative mechanism.

Indeed, in BMMCs the SCF-induced IKK2 activation is mediated by the PLCγ-Ca^2+^-Lyn-dependent phosphorylation of IKK2 on Y199. Huang *et. al*., 2003 described that additional to phosphorylation of the S177/S181-IKK2 motive, the SFK-dependent phosphorylation of the Y188/Y199-IKK2 motive is also critically involved in IKK2 activation [42–44]. Therefore, we speculate that the SCF-induced IKK2 activation also depends on the SFK-mediated Y199 phosphorylation. Interestingly, the SCF-induced phosphorylation of the S177/S181-IKK2 motive is transient whereas the phosphorylation of Y199-IKK persists up to 120min. This indicates that in contrast to the Y199-IKK2 phosphorylation, the phosphorylation of S177/S181-IKK2 is strongly regulated by phosphatases [54]. Interestingly, this suggests that the SCF-induced and long-persisting Y199-IKK2 phosphorylation primes IKK2 for additional stimulation with IL-33. Thereby, IKK2 phosphorylated on Y199 is targeted by the IL-33-induced signaling leading to increased IKK2 activity and therefore to potentiated mast cell effector functions.

Consistent with BMMCs, we also found a SFK-dependent IκBα phosphorylation in cells expressing constitutively active c-Kit. These data indicate that SCF in BMMCs, and constitutively active c-Kit, mediate IKK activation via SFKs.

The inefficiency of SCF or constitutively active c-Kit to induce IκB degradation leads to a model in which fewer IKK2 molecules are activated in response to c-Kit activation than in response to stimuli such as IL-33. Thus, the threshold of IKK activation which is necessary to induce IκBα degradation is not attained and therefore NF-κB still blocked.

Here, we also provide the mechanistical insight for this alternative IKK2 activation. IKK2 forms a complex with TAK1 and Lyn. This TAK1-IKK2-Lyn complex associates with c-Kit, via TAK1. The association of the TAK1-IKK2-Lyn complex with c-Kit activates Lyn which phosphorylates and activates IKK2. Since expression of the K63W-TAK1 mutant abrogated the association of the c-Kit-Lyn-TAK1-IKK2 complex, we hypothesize that TAK1 ubiquitinylation is essential to stabilize this complex and to mediate STAT3 activation. Which E3-Ligase mediates TAK1 ubiquitinylation is unknown. We presume a critical role of the E3-Ligase Cbl which is involved in c-Kit-mediated signaling in mast cells [55].

Importantly, in BMMCs and in HMC cells, the c-Kit-driven IKK activation also influences IL-33-induced signaling pathways, and therefore enables IL-33 to deploy its full biological effector functions. Therefore, the c-Kit-TAK1-IKK2 signaling not only mediates proliferation and survival, but also influences NF-κB mediated effector functions induced by TIR-family members.

These data show that the activated c-Kit-TAK1-IKK2 signaling module dramatically alters and enhances cellular effector functions. Therefore, dysregulation of the c-Kit-TAK1-IKK2 signaling module could induce pathological effector functions in inflamed tissues after infection, allergy and necrosis or supports tumor growth.

Given that activated c-Kit is involved in the development of autoimmune- [56, 57], and tumor-diseases [58–61], targeting of the c-Kit-TAK1-IKK2 signaling module might be an alternative approach to treat such diseases.

Especially the facts that **(i)** TAK1 or IKK2 inhibition induces cell death of tumor- but not of primary-mast cells [32] and that **(ii)** TAK1 or IKK2 inhibition overcomes the resistance of tumor cells to c-Kit inhibitors, suggest that targeting of the c-Kit-TAK1-IKK2 signaling module is a novel, specific and effective approach to therapeutically target pathogenic cellular effector functions.

## MATERIALS and METHODS

### Mice

Mice were maintained at the Animal Research Facility of the Universitätsklinikum Jena and Universität Frankfurt. Animal experiments were approved by the appropriate institutional and governmental committees for animal welfare. We used sex- and age-matched *myd88^−/−^*, *lyn^−/−^*, *ikk2^Δ^* [27], and either wild type (wt) littermates or *ikk2^F/F^*-mice (cre^−^ littermates) [27]. To induce deletion of IKK2, 6–8 week old ikk2^Δ^ or control mice (*ikk2^F/F^*; cre^−^ mice) were injected once with 250 μL of poly (I:C) (Sigma) i.p. and were analyzed after 21d. [27]

### Cell culture

HEK293T cells were cultured in DMEM (PAA) supplemented with 10% FCS, 100 U/ml penicillin, 100 mg/ml streptomycin (complete medium). HMC-1.1 and HMC-1.2 cells were cultured in complete RPMI (PAA). Ba/F3 clones were cultured in complete RPMI (PAA) supplemented with 20 ng/ml X63Ag-653 BPV-rmIL-3 supernatant (mIL-3). The ***NF-κB*** reporter mast cell line ***NF-κB***-EGFP-MC/9 (provided by Dr. E. A. Barbu, NIDCR, National Institutes of Health, Bethesda, Maryland, USA; [41]) was cultured in complete DMEM (PAA) supplemented with 20 ng/ml mIL-3. For generation of BMMCs, bone marrow cells were obtained from femur and tibia and were cultured in complete IMDM (PAA) supplemented with 20 ng/ml mIL-3. After 4 weeks cell culture consisted of 95% BMMCs (identified by expression of FcεRI, c-Kit, IL-33R).

### Transfection

HEK293T cells were transfected with pcDNA3.1 (empty vector), pcDNA3.1-c-Kit and/ or pCMV-HA-TAK1 or pCMV-HA-K63W-TAK1 (kindly provided by Prof. M. Kracht; Universität Giessen) by using lipofectamin (Invitrogen) according to the manufactural procedures.

### Cell stimulation and lysis

HEK293T cells were washed with ice cold PBS before lysis. HMC-1.1 and HMC-1.2 cells (10^6^ cells/ml) were treated with vehicle (DMSO) or inhibitors (60 min) (Merck Millipore). HMC-1.1 cells were then left unstimulated or were stimulated with IL-33 (peprotech). Ba/F3 cells, ***NF-κB***-EGFP-MC/9 cells or BMMCs (10^6^ cells/ml) were IL-3-starved (1 h). Afterwards, Ba/F3 cells were treated with DMSO or the IKK-inhibitor VII (60 min). MC/9 cells and BMMCs were DMSO- or inhibitor-treated (30 min) and were single (SCF or IL-33) or co-stimulated (SCF and IL-33) (peprotech). Cells were lysed (20 mM HEPES, pH7.5; 10 mM EDTA; 40 mM β-glycerophosphate; 2,5 mM MgCl_2_; 2 mM orthovanadate; 1 mM dithiothreitol; 20 μg/ml aprotinin; 20 μg/ml leupeptin supplemented with 1% Triton) and protein concentration was determined by using the BCA-kit (Pierce). Then protein samples were boiled in 6 × Laemmli buffer.

### Immunoprecipitation

Cell lysates from HEK293T or BMMCs (prepared with lysis buffer supplemented with 0,5% NP40) were incubated (overnight) with a Lyn-, c-Kit-, or TAK1-specific antibody (santa cruz) or goat-, mouse-, or rabbit-IgG as non-specific control immunoglobulins (Gentaur). Protein-G sepharose was added and incubated for 4 h. Precipitates were washed with lysis buffer and subsequently treated and boiled in 6 x Laemmli buffer.

### Western blotting

Samples were separated in 10% sodium dodecyl sulfate (SDS)-Laemmli gels and transferred by electroblotting onto nitrocellulose membranes (biostep). Membranes were blocked with dry milk and incubated (overnight) with antibodies detecting phosphorylated/ non-phosphorylated proteins or Hemagglutinin (HA)-tagged TAK1. We used the anti-HA antibody (provided by Prof. Böhmer, Centre of Medical Biomedicine, Jena), anti-pY719-c-Kit/c-Kit, anti-pY705-STAT3/STAT3, anti-pY694-STAT5/STAT5, anti-pS184/pS187-TAK1/TAK1, anti-pS176(IKK1)/pS-177(IKK2) or anti-pY199-IKK2 and anti-IKK1/2, anti-pS32-IκB*α*/IκBα, anti-pS473-PKB/Akt/PKB/Akt, anti-pY396-Lyn/Lyn, anti-Tubulin and anti-Ubiquitin [Cell Signaling; except anti-c-Kit, anti-TAK1, anti-IKK1/2, anti-Lyn and anti-Ubiquitin (Santa Cruz); anti-pY199-IKK2 and anti-pY396-Lyn (abcam) and anti-Tubulin (Sigma-Aldrich)]. Membranes were washed in 0.1% Tween/TBS and incubated with HRP-conjugated secondary antibodies: anti-rabbit-Ig, anti-goat-Ig (Santa Cruz) and anti-mouse-Ig (Thermo-scientific). Detection was performed using ECL reagent (Pierce).

### Flow cytometry

For detection of dead HMC-1.1 or HMC-1.2 cells, samples were washed with PBA (0.25% BSA; 0.02% sodium azide in PBS). Cells were left untreated or were treated with propidium iodid (PI). For detection of mast cell-specific surface marker, cells from cell cultures or from the peritoneal cavity were washed with PBA. Non-specific binding was blocked with anti-CD16/CD32 (clone 2.4G2) and rat-IgG (Jackson). Cells were stained with biotinylated IL-33R antibody (3E10; [62]) and PE- or APC-eFluor780-conjugated Streptavidin, PE-conjugated CD117 antibody (BioLegend) and FITC-conjugated FcεRI antibody (eBioscience). For determination of EGFP production, ***NF-κB***-EGFP- MC/9 cells (10^6^ cells/ml) were IL-3-starved (1 h) and stimulated with SCF or IL-33 (8 h). After harvesting and washing with PBA-buffer, cells were analyzed for EGFP production by using an LSR II flow cytometer (BD) and FlowJo 9.8.0 (Treestar Inc., Ashland, OR).

### ELISA and proliferation assays

HMC-1.1 and HMC-1.2 cells (10^5^ cells/ml) were treated with DMSO or the IKK-inhibitor VII. BMMCs (10^6^ cells/ml) were IL-3-starved (1 h) and pre-incubated (30 min) with vehicle (DMSO) or U-73122 (PLCγ inhibitor), BAPTA-AM (Ca^2+^ chelator), U0126 (MEK inhibitor), SP600125 (JNK inhibitor), the p38 inhibitor, SU6656 (SFK inhibitor), AG490 (Jak2 inhibitor), the IKK-inhibitor VII, the STAT3 inhibitor S3I-201 (all Merck Millipore) or Glivec^®^ (imatinib mesylat) (c-Kit inhibitor; kindly provided by Prof. Dr. Böhmer; Centre of Medical Biomedicine, Jena). BMMCs were stimulated with SCF, IL-33 or both. BaF/3 cells (2,5 × 10^5^ cells/ml) were IL-3-starved (1 h) and treated with DMSO or the IKK-inhibitor VII. For proliferation assays cells were cultured for 54 h. [3H]-thymidine (1 μCi) in 25 μl complete IMDM (PAA) (without IL-3) was added to each well for additional 18 h. Incorporated radioactivity was measured by using a *β*-scintillation counter (Perkin Elmer, Rodgau-Jügesheim, Germany). For ELISA experiments supernatants were collected (after 8 h) and analyzed for cytokine production using matched pair antibodies (ebioscience).

### Determination of cell death

HMC-1.1 and HMC-1.2 cells were treated with the IKK-inhibitor VII (48 h), harvested, washed (PBA-buffer) and treated with propidium iodide (PI). Cells were analyzed with the LSR II flow cytometer (BD) and FlowJo 8.1.1 (Treestar Inc.).

### Ca^2+^ mobilization

For calcium assays, the FLIPR Calcium 4 Assay Kit and the FlexStation3 microplate reader (Molecular Devices, Sunnyvale CA) were used. All recordings were done on PLL coated 96 well plates (Greiner). 1 × 10^5^ BMMCs per well were resuspended in 80 μl Phenol Red-free DMEM (Life Technologies), were mixed with 20 μl Ringer's solution (80 mM KCl; 130 mM NaCl; 1 mM MgCl_2_; 1 mM CaCl_2_; 10 mM HEPES pH7.3; 20 mM Glucose; Probenecid 25 mM) and an equal volume of loading dye. Prior to stimulation with SCF cells were pre-treated with inhibitors (30 min).

### Histology

Samples of back skin or ear pinna were fixed in 4% (v/v) buffered formalin and embedded in paraffin ensuring a cross-sectional orientation. For detection of mast cells, 5 μm sections were metachromatically stained (Giemsa). Mast cell numbers were counted per ear or back skin section over a length of 1 cm. Mean of 3 sections per mouse was calculated ± SD.

### Statistical analysis

All experiments were performed at least three times. Proliferation assays, and ELISAs were performed at least in a 6-fold determination. Cytokine concentration, stimulation indices (SI) and mast cell numbers per peritoneum are indicated as the mean of measurements ± standard error of the mean (SEM). The statistical analysis was performed with IBM SPSS Statistics version 21.0 (IBM, Ehningen, Germany). Statistical significance was assessed by students *T*-test. Statistical significance was accepted for *p* < 0,05 (*, *p* < 0.05; **, *p* < 0.01; ***, *p* < 0.001).

## SUPPLEMENTARY FIGURES


