# Supplementary material for: TAK1 and IKK2, novel mediators of SCF-induced signaling and potential targets for c-Kit-driven diseases
Source: Oncotarget. 2015 Sep 1;6(30):28833–50. doi: 10.18632/oncotarget.5008 (PMC4745695; doi:10.18632/oncotarget.5008)
Supplement: Supplementary file 1 [file oncotarget-06-28833-s001.pdf]

SUPPLEMENTARY FIGURES

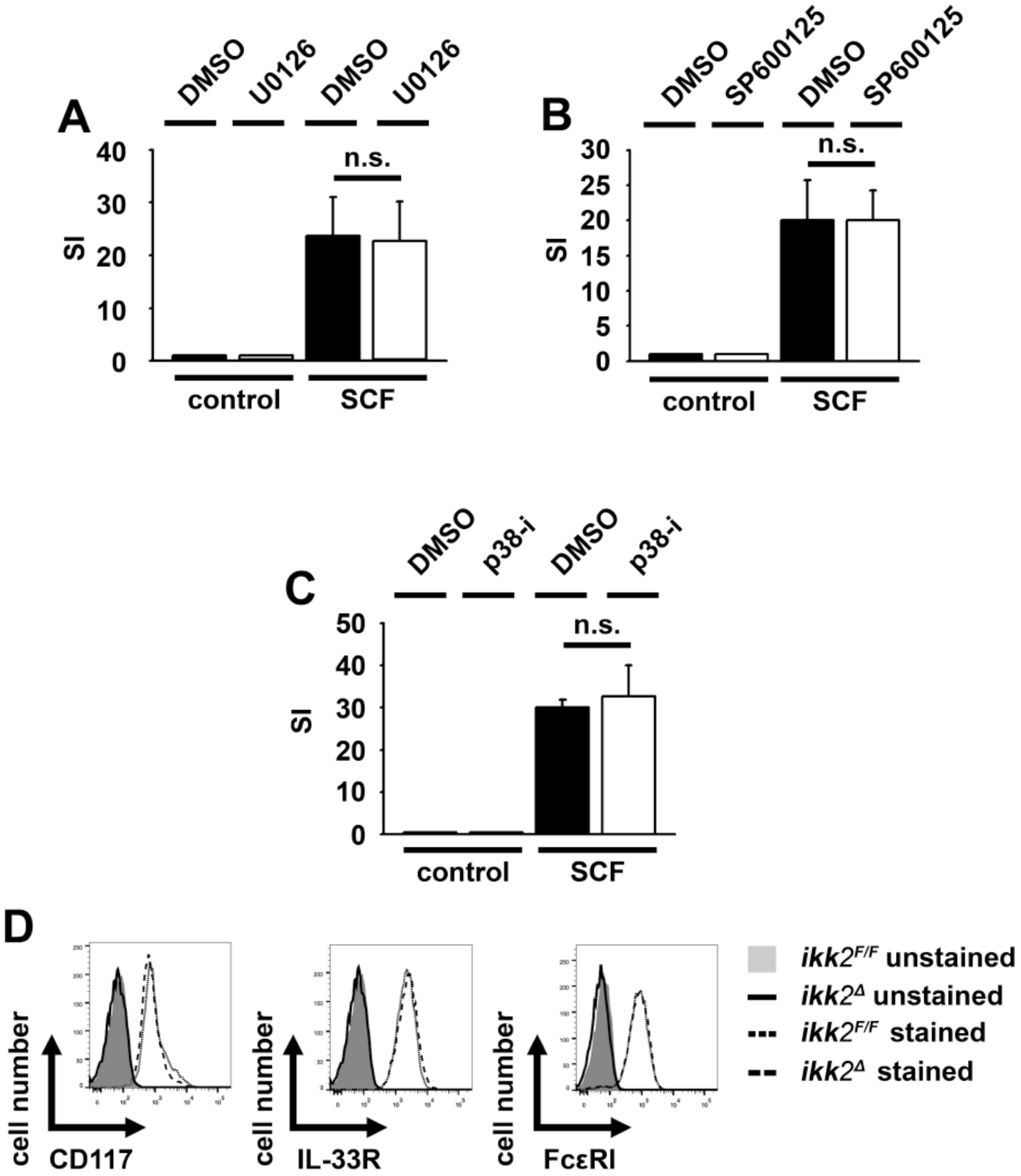

Supplementary Figure S1: SFKs are crucial for SCF-induced IKK2 activation. **A–C.** BMMCs were pre-treated with DMSO (vehicle) or U0126 (A), SP600125 (B) or the p38 inhibitor (C) (all 5 µM). Cells were stimulated with SCF (50 ng/ml) and were probed with [H^3]-thymidine and analyzed by β-counting. **D.** *Ikk2$^{F/F}$* or *ikk2$^{\Delta}$* BMMCs were analyzed for the surface expression of c-Kit (CD117), IL-33R and FcεRI by flow cytometry. (*Continued*)

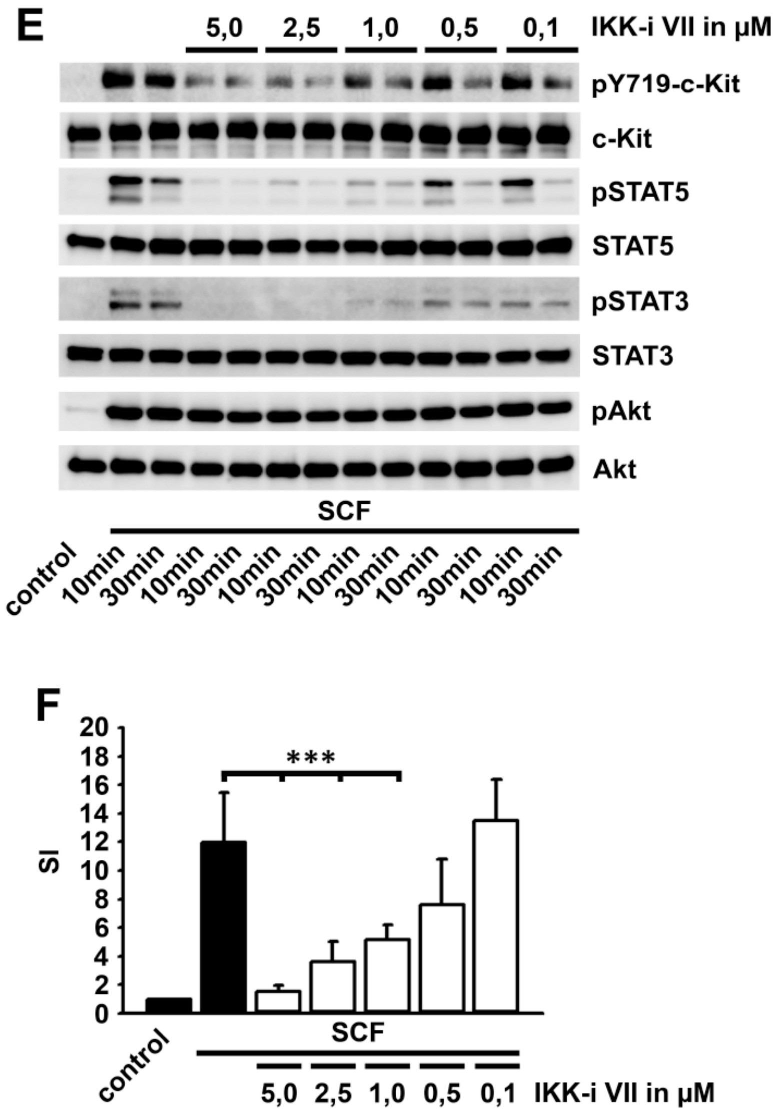

Supplementary Figure S1: (*Continued*) SFKs are crucial for SCF-induced IKK2 activation. E, F. BMMCs were treated with the IKK-inhibitor VII (IKK-i VII) and were stimulated with SCF. Lysates were analyzed by western blotting (E) or cells were probed with [H^3]-thymidine and analyzed by β-counting (F).

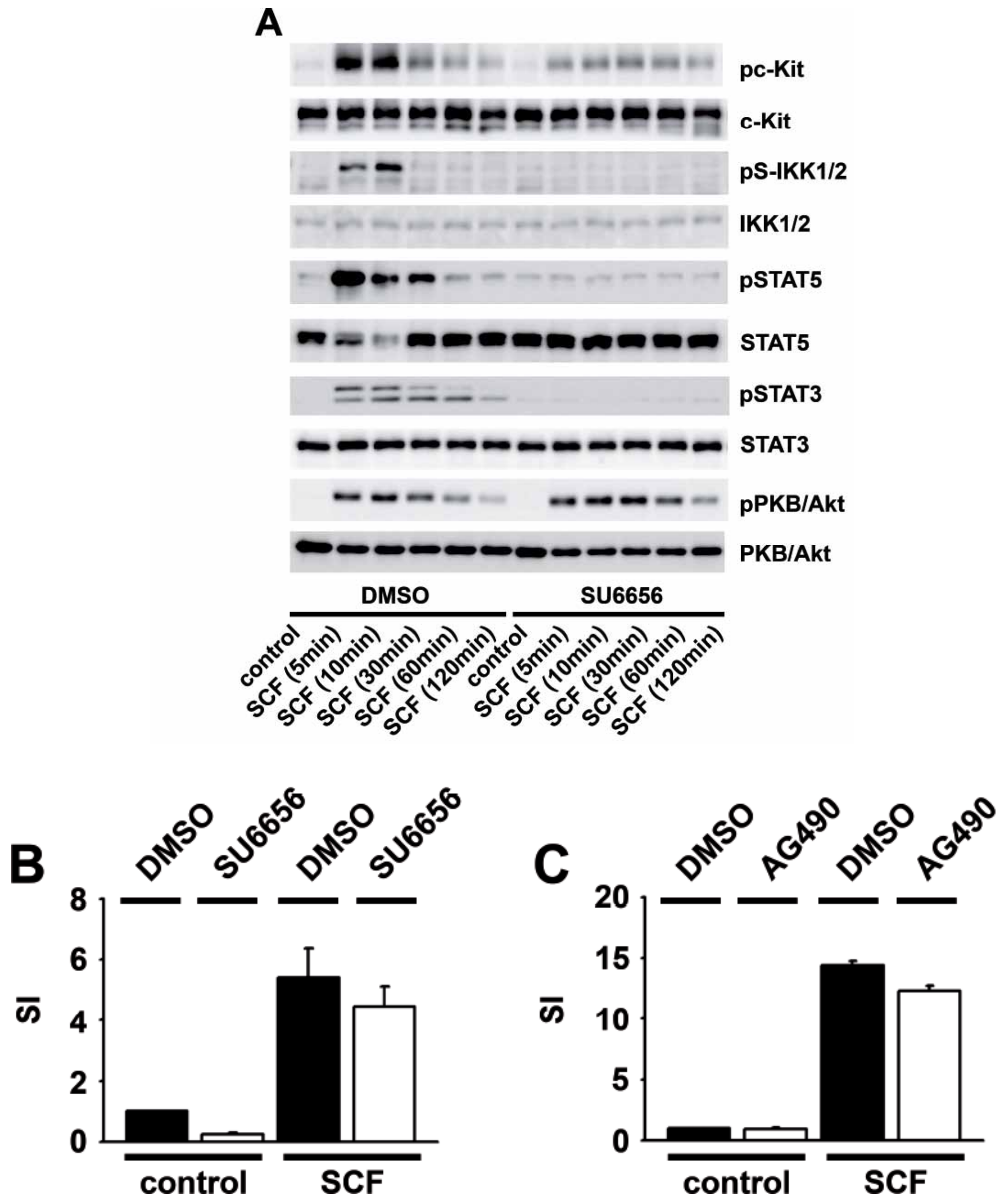

Supplementary Figure S2: The SFK, Lyn regulates SCF-induced signaling. A, B. BMMCs were pre-treated with DMSO (vehicle) or SU6656 (5 µM) and stimulated with SCF (50 ng/ml). Lysates were analyzed by western blotting (A) or cells were probed with [H^3]-thymidine and analyzed by β-counting (B). **C.** BMMCs were pre-treated with DMSO (vehicle) or AG490 (5 µM) and stimulated with SCF (50 ng/ml). Cells were probed with [H^3]-thymidine and analyzed by β-counting. (*Continued*)

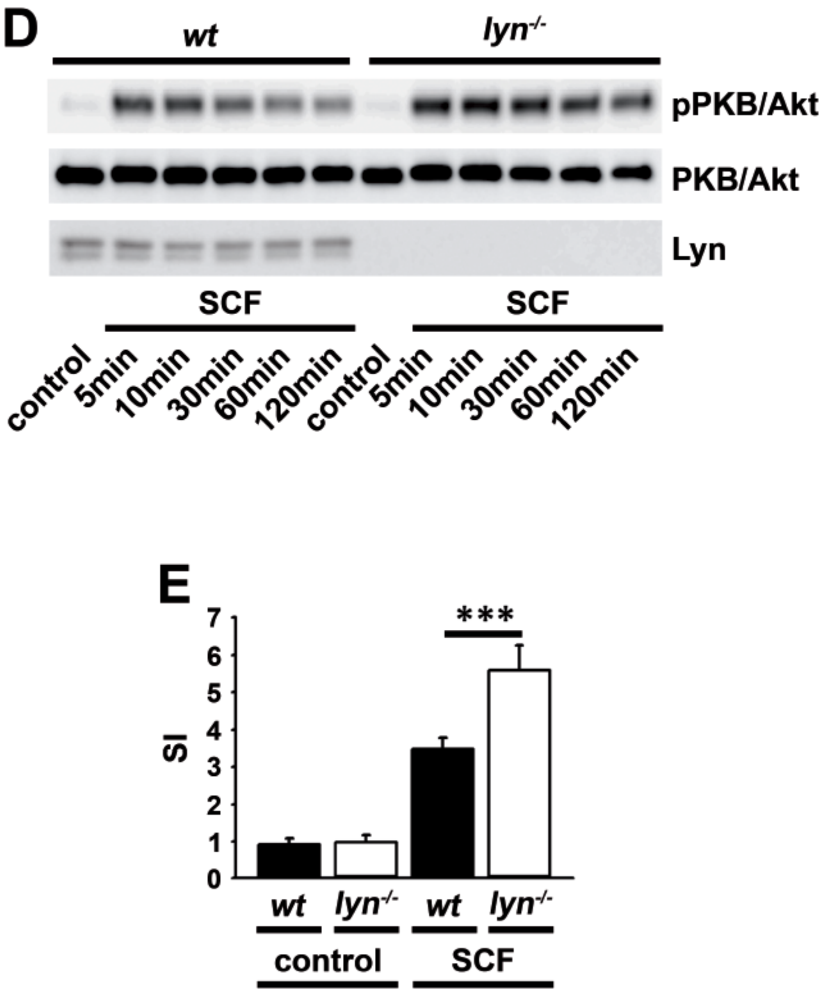

Supplementary Figure S2: (*Continued*) The SFK, Lyn regulates SCF-induced signaling. D, E. Wt and *lyn*$^{-/-}$ BMMCs were stimulated with SCF (50 ng/ml) and lysates were analyzed by western blotting (D) or cells were probed with [H^3]-thymidine and analyzed by β-counting (E).

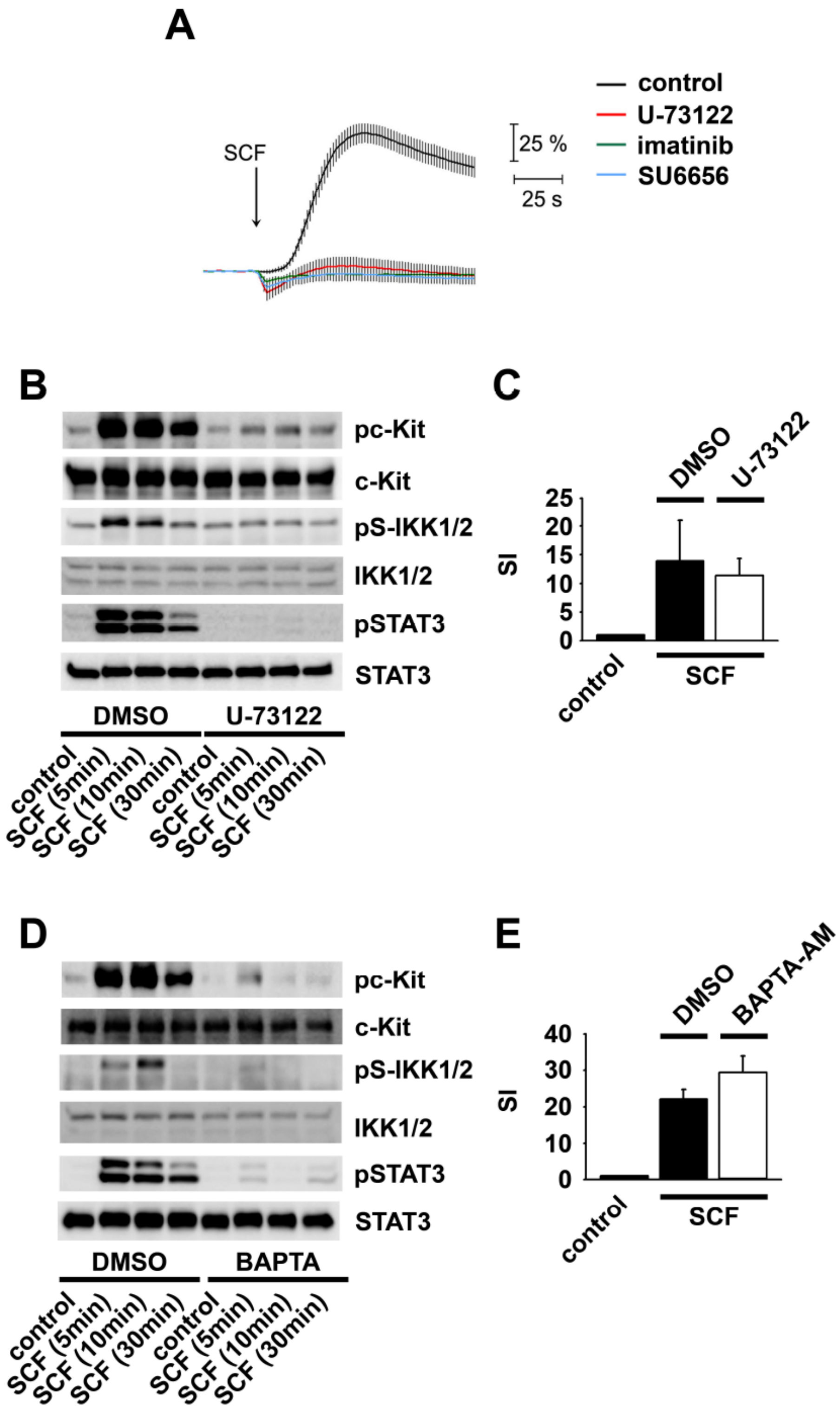

Supplementary Figure S3: Ca^{2+} mobilization mediates the SCF-induced activation of the c-Kit-IKK2-STAT signaling pathway. A. BMMCs were pre-treated with DMSO (vehicle) or U-73122, imatinib or SU6656 (all 5 μM). Cells were stimulated with SCF and Ca^{2+} mobilization was determined. **B–E.** BMMCs were pre-treated with DMSO (vehicle) or U-73122 (B, C) or BAPTA-AM (D, E) (both 5 μM). Cells were stimulated with SCF (50 ng/ml) and lysates were analyzed by western blotting (B, D) or cells were probed with $[H^3]$-thymidine and analyzed by β-counting (C, E). (*Continued*)

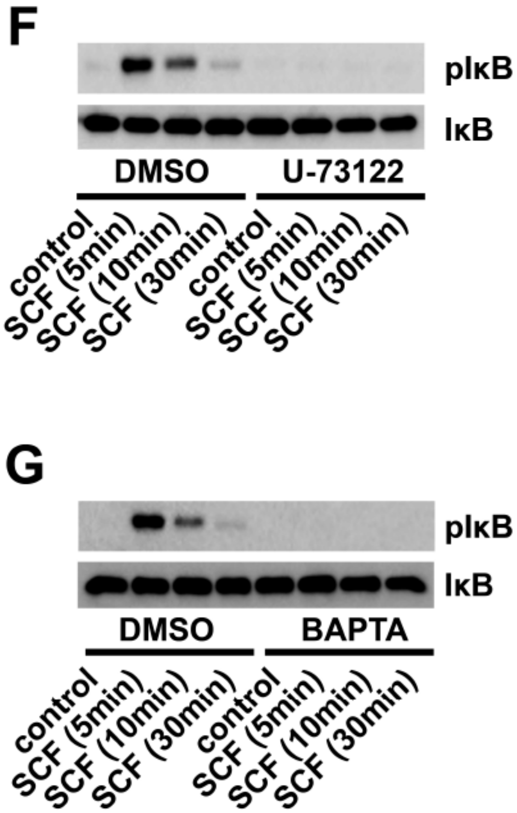

Supplementary Figure S3: (*Continued*) Ca^{2+} mobilization mediates the SCF-induced activation of the c-Kit-IKK2-STAT signaling pathway. F, G. BMMCs were pre-treated with DMSO (vehicle) or U-73122 (F) or BAPTA-AM (G) (both 5 µM). Cells were stimulated with SCF (50 ng/ml) and lysates were analyzed by western blotting.

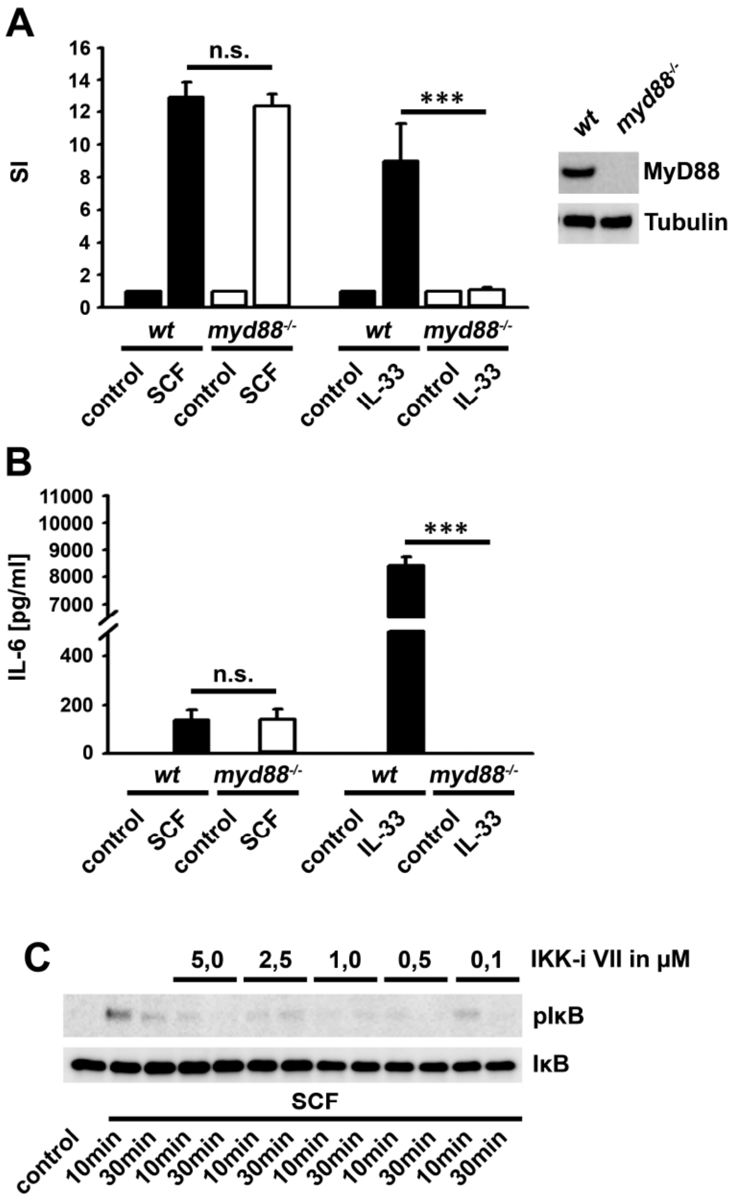

Supplementary Figure S4: MyD88 is important for IL-33-induced but not SCF-induced effector functions. A. Wt or *myd88*$^{-/-}$ BMMCs were stimulated with SCF or IL-33 (both 50 ng/ml). Cells were probed with [H^3]-thymidine and analyzed by β-counting (A) or supernatants were collected and analyzed for IL-6 production by ELISA **B. C.** BMMCs were pre-treated with DMSO (vehicle) or the IKK-inhibitor VII (as indicated) and stimulated with SCF (50 ng/ml). Lysates were analyzed by western blotting.

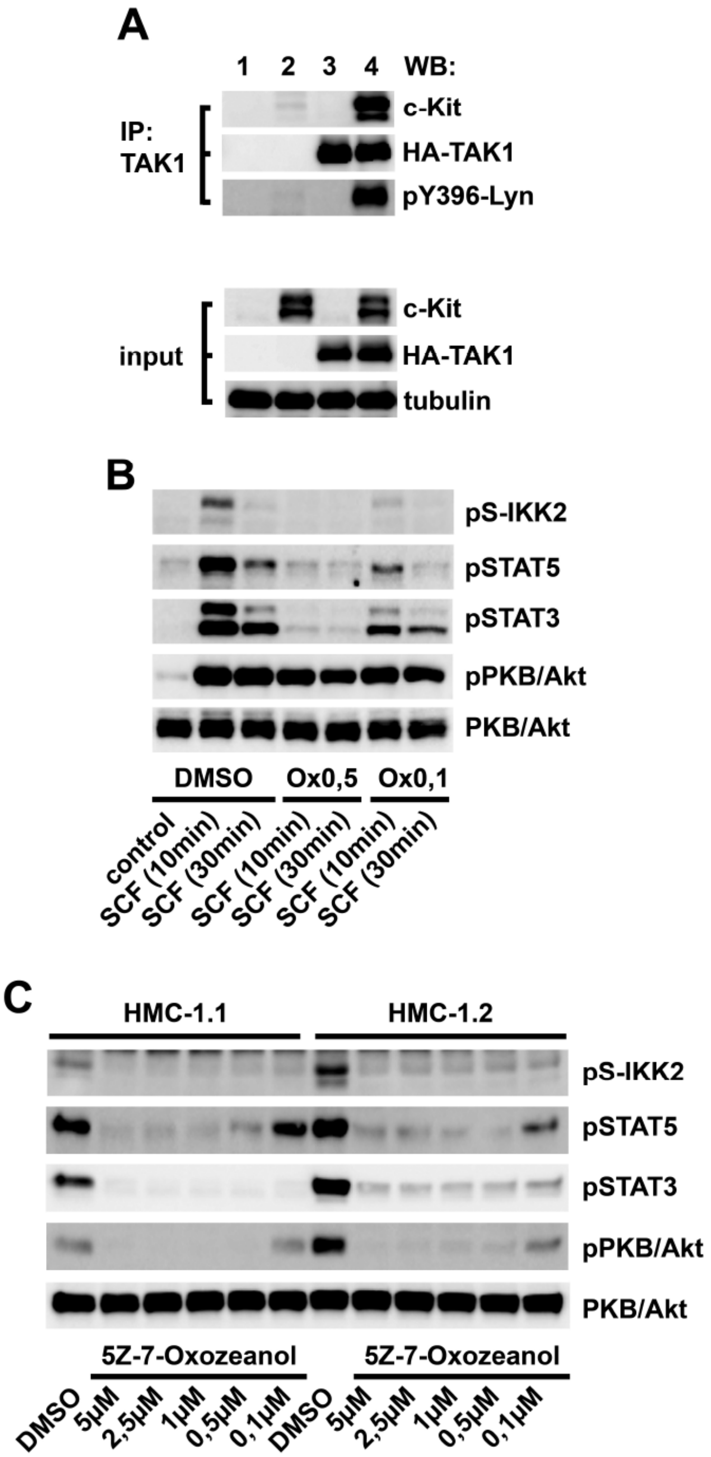

Supplementary Figure S5: The SCF-induced activation of the c-Kit-IKK2-STAT signaling depends on TAK1. A. HEK293T cells were transfected with pcDNA3.1 (empty vector) (lane 1), pcDNA3.1-c-Kit (lane 2), pCMV-HA-wt-TAK1 (lane 3) or pcDNA3.1-c-Kit and pCMV-HA-wt-TAK1 together (lane 4). Lysates were subjected to a TAK1-specific immunoprecipitation and the precipitates were analyzed by western blotting. Total cell lysates were analyzed for the equal expression of the transfected proteins by western blotting. **B.** BMMCs were treated with the TAK1 inhibitor 5Z-7-oxozeanol (Ox.). Cells were stimulated with SCF and lysates were analyzed by western blotting. **C.** HMC-1.1 or HMC-1.2 cells were treated with the TAK1 inhibitor 5Z-7-oxozeanol and lysates were analyzed by western blotting.

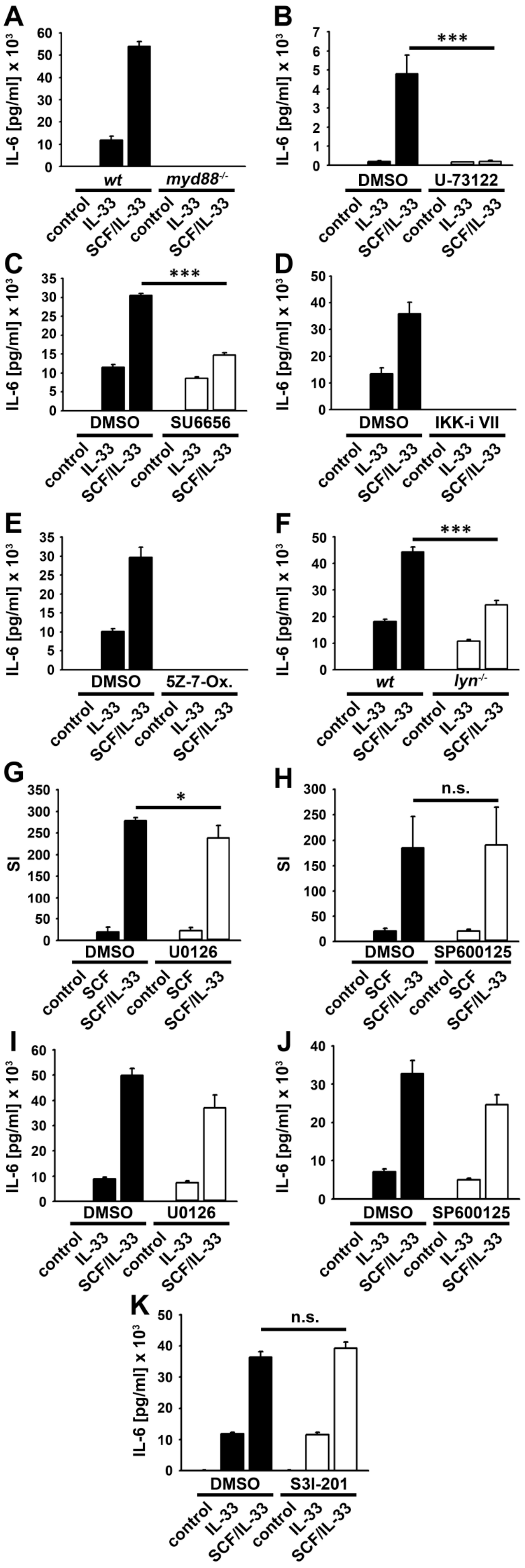

Supplementary Figure S6: Co-stimulation with SCF and IL-33 induced MyD88-, Lyn- and IKK2-dependent cytokine production. A–K. Wt BMMCs treated with DMSO (vehicle) or different inhibitors or from different knock out mice strains [*myd88*$^{-/-}$ (A); *lyn*$^{-/-}$ (F)], were stimulated with SCF or IL-33 alone or SCF in combination with IL-33 (both 50 ng/ml). Co-stimulation was performed by pre-incubation of the cells with SCF (30min) and the subsequent stimulation with IL-33. Cells were analyzed for IL-6 production by ELISA (A–F, I–K) or were probed with [H^3]-thymidine and analyzed by β-counting (G, H).
